# Electron-Phonon Coupling Parameter of Ferromagnetic Metal Fe and Co

**DOI:** 10.3390/ma14112755

**Published:** 2021-05-23

**Authors:** Kyuhwe Kang, Gyung-Min Choi

**Affiliations:** 1 Department of Energy Science, Sungkyunkwan University, Suwon 16419, Korea; chrisdior@skku.edu; 2 Center for Integrated Nanostructure Physics, Institute for Basic Science (IBS), Suwon 16419, Korea

**Keywords:** electron-phonon coupling, time-resolved spectroscopy, nanoscale thermal transport

## Abstract

The electron-phonon coupling (*g*) parameter plays a critical role in the ultrafast transport of heat, charge, and spin in metallic materials. However, the exact determination of the *g* parameter is challenging because of the complicated process during the non-equilibrium state. In this study, we investigate the *g* parameters of ferromagnetic 3*d* transition metal (FM) layers, Fe and Co, using time-domain thermoreflectance. We measure a transient increase in temperature of Au in an FM/Au bilayer; the Au layer efficiently detects the strong heat flow during the non-equilibrium between electrons and phonons in FM. The *g* parameter of the FM is determined by analyzing the temperature dynamics using thermal circuit modeling. The determined *g* values are 8.8–9.4 × 10^17^ W m^−3^ K^−1^ for Fe and 9.6–12.2 × 10^17^ W m^−3^ K^−1^ for Co. Our results demonstrate that all 3*d* transition FMs have a similar *g* value, in the order of 10^18^ W m^−3^ K^−1^.

## 1. Introduction

The ultrafast photoexcitation, triggered by femtosecond laser pulse, of metallic surfaces has enabled the investigation of the thermal relaxation process between excited electrons and phonons in metals. In particular, thermoreflectance, which refers to the change in reflectivity with temperature, has been used to collect the time-resolved temperature response on metal surfaces [1]. A key parameter for the ultrafast thermal relaxation in metals is the electron–phonon coupling (*g*), which has been reported for several materials [2,3,4,5,6,7,8]. This parameter has been studied for ferromagnetic 3*d* transition metals (FMs) to understand the ultrafast dynamics of magnetization. The most heavily studied FM is Ni, and the *g* parameter of Ni has been reported to be 8 and 10.5 × 10^17^ W m^−3^ K^−1^, determined from the transient temperature excursion in a single Ni layer [9,10]. However, the exact determination of the *g* parameter from the temperature responses of the FM is often challenging because of multiple couplings among electrons, phonons, and magnons. In a prior work, we demonstrated that the *g* parameter can be accurately determined by measuring the transient heat current from Ni to Au in a Ni/Au bilayer, and determined the *g* parameter of Ni to be 8.6 × 10^17^ W m^−3^ K^−1^ [11]. In this study, we investigate the *g* parameters of two FM materials, Fe and Co, by measuring the transient heat flow in an FM/Au bilayer. As far as we know, there is no experimental report of the *g* parameter of Fe, and theoretical estimation shows a large deviation for *g* of 7–55 × 10^17^ W m^−3^ K^−1^ [8,12,13,14]. Previously, the *g* parameter of Co was reported to be 20 × 10^17^ W m^−3^ K^−1^, but this value is obtained with a complicated structure and multiple fitting parameters [15]. Theoretical estimation of the *g* parameter of Co is 7 and 33 × 10^17^ W m^−3^ K^−1^ [13,14].

To investigate the *g* parameters of the target materials, we employ an indirect optical heating configuration: pump and probe beams hit the opposite surface of the sample. When the pump beam hits the bottom surface of the metallic bilayer, a transient heat flow occurs and leads to temperature dynamics on the top surface, which can be measured by the probe beam [11,16,17]. Indirect heating has several advantages over direct optical heating (pump and probe beams on the same surface). It has less contribution from non-thermalized electrons to the signal. In addition, as the pump and probe are spatially separated, they are free from several optical artifacts that occur when the pump and probe beams are spatially overlapped. In this study, we adopted both direct and indirect optical heating configurations to determine the *g* parameters of the target FM materials. In both configurations, we measure the Au temperature instead of the FM temperature; the probe beam is fixed on the Au surface, while the pump beam is either on the FM or Au surface. Au is known to have a small value of *g*, and hence the *g* of Au has only a minor effect on the transient heat flow at timescales of a few tens of picoseconds. Thus, the transient temperature excursion of the Au layer is sensitive to the *g* parameter of FM. In addition, the interpretation of the temperature of Au is simpler than that of FM, because Au does not have magnons.

To analyze the time-variant temperature excursion, we constructed a thermal circuit model based on a two-temperature model (TTM) in one-dimensional heat flow [11,17]. This circuit model is more convenient than a continuum analytical model in describing the initial distribution of thermal energy, the depth profile of temperature response, and temperature dependence of the thermodynamics parameters. By fitting the experimental results with the thermal circuit modeling, we determined the *g* parameters of 8.8–9.4 × 10^17^ for Fe and 9.6–12.2 × 10^17^ for Co, with a unit of W m^−3^ K^−1^. The *g* values have relative errors of 7% and 24%, respectively, for Fe and Co, depending on the heating configurations (direct and indirect). The reason for this discrepancy could be the uncertainties in the thermal modeling and experiment.

## 2. Materials and Methods

We prepared two types of the bilayer samples: sapphire substrate (sap)/FM 20 nm/Au 100 nm, where the FM is Fe or Co. The samples were fabricated with a magnetron sputtering system, with a process pressure of 3 mTorr and base pressure of less than 10^−7^ Torr. X-ray reflectometry was used to measure the thickness of each metallic film. All layers were deposited at room temperature.

To observe the dynamics of the temperature of the samples, we employed an optical pump-probe technique called time-domain thermoreflectance (TDTR). Pulsed pump and probe beams were generated by a Ti-sapphire laser oscillator with a repetition rate of 80 MHz. Both beams had a wavelength of 785 nm of a wavelength and pulse width, full width a half maximum (FWHM), of 0.1 ps; however, the pulse width of the pump beam was extended to 1 ps through an electro-optic modulator that modulated the pump beam at a frequency of 9.5 MHz. In addition, the probe beam was modulated at 200 Hz via an optical chopper. We collected the TDTR signal that had two frequency components of 9.5 MHz and 200 Hz to suppress the noise level and reject signals caused by pump leaking to the detector.

TDTR collects reflection changes (∆*R*) of the probe beam from the sample surface, depending on the time delays between the incidence of the pump beam and the probe beam on the sample. ∆*R* is typically expressed as a linear combination of changes in both the electron and phonon temperatures (∆*T*_e_ and ∆*T*_ph_) [4]. In this work, we collected TDTR data from only the surface of the Au layer; ∆*T*_ph_ of the Au layer has a dominant contribution to ∆*R*, while the role of ∆*T*_e_ is minor [17]. The ∆*T*_e_ effect on ∆*R* can be significant during a few ps after the pump irradiation, when ∆*T*_e_ becomes much higher than ∆*T*_ph_. After a few ps, when the peak of ∆*T*_e_ disappears, ∆*R* from the Au surface is dominated by ∆*T*_ph_ [18,19,20]. We used the ratio of the in-phase signal (*V*_in_) and out-of-phase signal (*V*_out_) as the temperature response. The ratio *V*_in_/*V*_out_ makes the TDTR signal more robust due to the following two issues: the defocus of the pump beam and changes in the spatial overlap between the pump and probe beams on the sample surface [21,22,23]. Both issues are caused by the change in the pump beam pathway length to adjust the time delay between the pump and probe beams. The motion of a delay stage causes a slight spatial defocus and misalignment in the pump spot on the sample. The ratio *V*_in_/*V*_out_ suppresses the error associated with these issues because both *V*_in_ and *V*_out_ have the same dependencies on defocus and overlap.

## 3. Results

### 3.1. Overview

**Figure 1:**
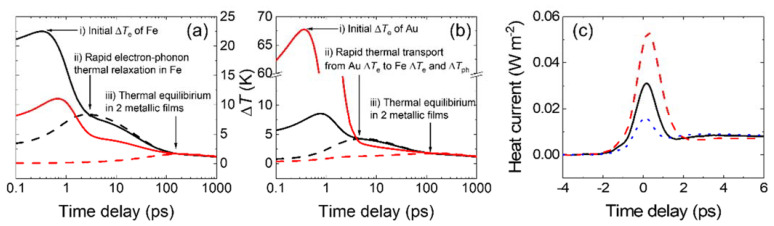
Analytic results of a thermal circuit simulation for the sap/Fe 20 nm/Au 100nm sample. An initial optical pump pulse is given on the Fe side (**a**) or Au side (**b**). The black solid/black dashed/red solid/red dashed line is ∆*T*_e_ of Fe/∆*T*_ph_ of Fe/∆*T*_e_ of Au/∆*T*_ph_ of Au, respectively. Three arrows indicate the timescales for (i) the thermalization in the electron bath (a few hundreds of femtoseconds), (ii) the thermal relaxation between the electron and phonon baths in Fe (**a**) or the spatial electronic heat transport from Au into Fe, which subsequently flows fast to the Fe phonon bath (**b**) (a few picoseconds), and (iii) the thermal relaxation between Fe and Au layers (a few hundreds of picoseconds). (**c**) The heat current per unit area from Fe to Au is driven by the optical excitation of Fe. The black solid/red dashed/blue dotted line is a simulation result with 8.8/4.4/17.6 × 10^17^ W m^−3^ K^−1^ of the *g* parameter of Fe. The larger the *g* parameter is, the smaller the heat current that flows from Fe to Au.

In this section, we introduce several thermal interaction steps in the bilayer samples according to the time scales. When the optical pump is projected onto the samples, the optical energy excites the electrons first, and the higher electronic energy is thermalized at 100 fs timescale, so that we can define ∆*T*_e_. Then, thermalization between the electron bath, with a small heat capacity of *c*_e_, and phonon bath, with a much larger heat capacity of *c*_ph_, occurs in a few ps timescales. In particular, in this sample structure, the electron-phonon thermal relaxation is much faster in the FM layer than in the Au layer because of the large difference in the values of *g* for FM and Au. Finally, the phonon bath of Au is thermally equilibrated with the phonon bath of FM at around 100 ps timescale. These processes are shown in Figure 1a,b, where the thermal model predicts the temperature dynamics of the Fe/Au bilayer with initial pump energy on the Fe side and the Au side, respectively.

To determine the *g* parameter of FM, we focused on $∆T_{\mathrm{ph}}$ of the Au layer (∆*T*_ph,Au_), which is more sensitive than that of the FM layer (∆*T*_ph,FM_) to the change in the *g* parameter of FM. This is because the ultrafast heat transport from the FM electron bath to the Au layer at short timescales is critically dependent on the magnitude of the electron-phonon thermal interaction in the FM layer. Figure 1c shows that the magnitude of *g* of FM determines the amount of initial electronic heat flow from FM to Au. This mechanism allows us to reliably determine the *g* parameter of the FM using the FM/Au bilayer.

### 3.2. Thermal Circuit Model

For the thermal circuit model based on TTM, we used Equation (1) to reproduce the TDTR data analytically.

(1) $$c_{e}\frac{dT_{e}}{dt}=g\left(T_{\mathrm{ph}}-T_{e}\right)+\Lambda_{e}\frac{\partial^{2}T_{e}}{\partial x^{2}}+p\left(x,t\right),\;\;c_{\mathrm{ph}}\frac{dT_{\mathrm{ph}}}{dt}=g\left(T_{e}-T_{\mathrm{ph}}\right)+\Lambda_{\mathrm{ph}}\frac{\partial^{2}T_{\mathrm{ph}}}{\partial x^{2}},$$

where the subscripts e and ph represent electron and phonon baths, respectively, *T*_j_ is the temperature of the bath j, *Λ*_j_ is the thermal conductivity of the bath j, and *p* is the initial electronic thermal energy absorbed from the optical pump pulse. The electron properties of *c*_e_ and *Λ*_e_ are expressed as linear functions of *T*_e_, while the phonon properties of *c*_ph_ and *Λ*_ph_ are constant values in the model.

**Figure 2:**
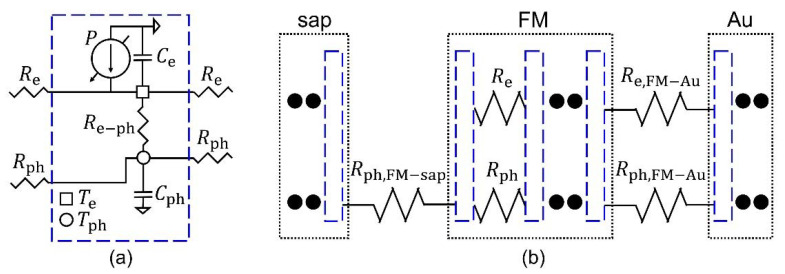
(**a**) The sub-circuit in the thermal circuit model, as indicated with a blue dashed line. The square/circle node has $∆T_{e}$/$∆T_{\mathrm{ph}}$ information and is connected to another square/circle nodes in neighboring sub-circuits. The heat source *P* is connected to the square node. (**b**) A schematic of the whole circuit, including the interfacial thermal resistances between two sub-circuits of different materials.

The thermal circuit model consists of several discrete sub-circuits that have a specific length, such as the finite element method, as shown in Figure 2a,b. Each sub-circuit has two types of heat capacitance of electron and phonon baths (*C*_e_ and *C*_ph_), and they are connected through an electron-phonon thermal coupling resistance (*R*_e-ph_). In addition, the heat capacitors in neighboring sub-circuits in a single material that have the same types of carrier baths (electrons or phonons) are connected with a spatial thermal resistance (*R*_e_ or *R*_ph_). At the interface between the different materials, the neighboring sub-circuits are connected by interfacial thermal resistances (*R*_j,FM-i_). *R*_j,i-FM_ is given according to the type of carrier (j) and material (i). These circuit elements are described with intrinsic material properties, finite spatial heights (*h*), and areas (*A*), as follows:

(2) $$C_{j}=c_{j}Ah,\;R_{e-\mathrm{ph}}=1/\left(gAh\right),\;R_{j}=h/\left(A\Lambda_{j}\right),\;R_{j,\mathrm{FM}-i}=1/\left(AG_{j,\mathrm{FM}-i}\right),$$

where j is e or ph, and i is Au or sap for each FM-Au interface or FM-sap interface. We calculate *R*_e,FM-Au_ with the Wiedemann-Franz law using the reported values of interfacial electrical resistance. The resistance for the Co-Au interface is assumed to be the same as the reported resistance for the Co-Cu interface, while that of the Fe-Au interface is chosen as the mean value of resistances for Co-Cu and Ni-Cu interfaces [24]. *R*_ph,FM-i_ for the FM-Au interface is chosen as the same values as that for the Ni-Au interface [25], and that for the FM-sap interface is determined in this work at a time range after 200 ps, where the heat flow from the metal layers to the sapphire substrate dominates the TDTR response.

**Figure 3:**
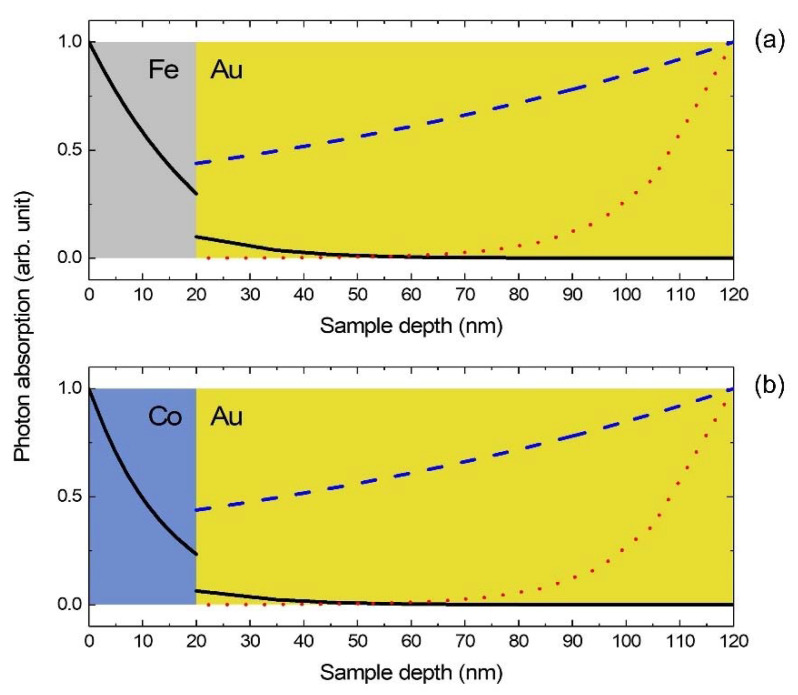
Initial electronic thermal energy distributions in the Fe/Au sample (**a**) and Co/Au sample (**b**). The black solid lines are that with the pump on the FM side, which are same with the optical energy absorption by the sample. Meanwhile, the blue dashed lines are that with the Au side pump, which are calculated with the phenomenological penetration depth of optically excited non-thermal electrons in Au, adapted from Ref. [27]. The red dotted lines are weight functions of ∆*T*_ph_ in all Au sub-circuits to the TDTR signal.

The heat transport in the FM/Au bilayer is driven by the energy sources (*P*) on the electron nodes of the metallic sub-circuits. This *P* represents the initial electronic thermal energy obtained from optically excited electronic energy with a finite spatial dimension ($P=A\int_{x_{0}}^{x_{0}+h}p\;dx$). For the optical pump on the FM side, we assume that *P* has a Gaussian form of 1 ps FWHM, which is the same as the pulse width of the pump beam. This assumption is reasonable, given that the time for thermalization of photoexcited electrons is approximately a few tens of femtoseconds in FM [26]. Thus, we calculated *P* using an optical transfer matrix method with the reported refractive index (*n*) values, assuming that electronic thermalization occurs instantaneously with the photoexcitation. On the other hand, Hohlfeld et al. reported that photoexcited electrons in Au move ballistically over 100 nm, and hence the electrons are thermalized spatially far from the photoexcited area [27]. The ballistic motion has been understood by the Fermi velocity of non-thermal electrons [28]. For the optical pump on the Au side, we followed this research and applied a phenomenological penetration depth of optically excited thermal electrons, i.e., 121.3 nm. The calculated *P* are described in Figure 3a,b. The fluence of the optical pump pulse energy was adjusted to be 0.65 J m^−2^ in all the experiments.

The reflected probe beam also has a penetration depth, and hence the TDTR data are composed of a linear combination of ∆*T*_ph_ of the sub-circuits. To reproduce the penetration depth effect in the thermal model, proper weights of ∆*T*_ph_ in all sub-circuits to the total TDTR signal are necessary. A transfer matrix method was used again, with reported information on the temperature-dependent refractive index (d*n*/d*T*) for Au [29]. The weight functions for ∆*T*_ph_ along the depth from the Au surface is shown in Figure 3. The intrinsic thermal properties for the thermal circuit model are summarized in Table 1.

### 3.3. Fitting Results

**Figure 4:**
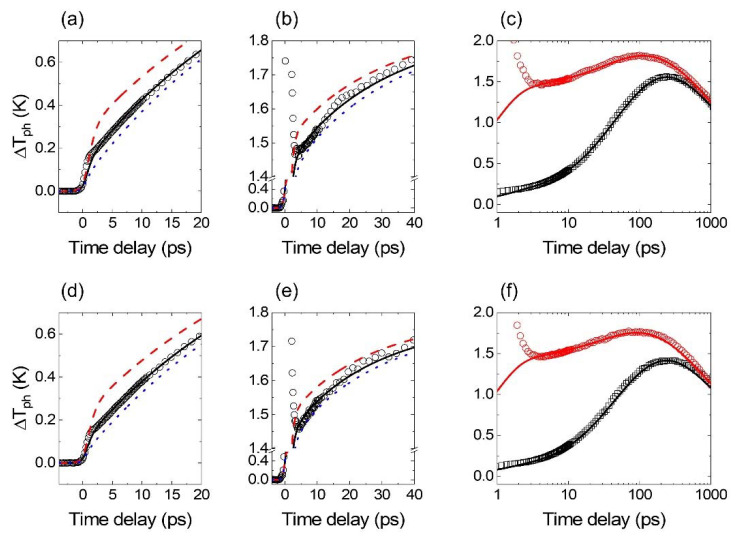
Fitting results between the thermal circuit simulation and TDTR data (all symbols). (**a**) and (**b**) ∆*T*_ph_ of the Au layer in the Fe/Au sample with a pump pulse incidence on the Fe side and the Au side, respectively. The black circles are TDTR results. The black solid/red dashed/blue dotted lines are the thermal circuit model results with (**a**) 8.8/4.4/17.6 and (**b**) 9.4/4.7/18.8 of the *g* parameter of Fe. (**c**) Overall excursion of ∆*T*_ph_ of the Au during 1 ns. The black squares/red circles are TDTR results with the indirect/direct heating. The black/red line is the thermal model results for the indirect/direct heating with the *g* parameter of Fe of 9.1. (**d**–**f**) Same configurations as above, but the Co/Au sample is analyzed. The black solid/red dashed/blue dotted lines are the thermal circuit model results with (**d**) 9.6/4.7/19.2 and (**e**) 12.2/6.1/24.4 of the *g* parameter of Co. The *g* parameter of 10.9 of Co is used for black and red lines in (**f**). All *g* parameters are in a unit of 10^17^ W m^−3^ K^−1^.

To determine a reliable value of the *g* parameter, we perform fitting processes separately with two types of optical configurations: the optical pump on the FM side (indirect heating) and on the Au side (direct heating). The determined *g* parameter of Fe has 7% deviation, depending on the heating configuration: 8.8 × 10^17^ W m^−3^ K^−1^ for the indirect heating and 9.4 × 10^17^ W m^−3^ K^−1^ and for the direct heating. The *g* parameter of Co is determined with 24% deviation: 9.6 × 10^17^ W m^−3^ K^−1^ for the indirect heating and 12.2 × 10^17^ W m^−3^ K^−1^ for the direct heating. For both Fe and Co, the direct heating configuration results in a higher value of the *g* parameter compared to that with the indirect heating configuration. The deviation in *g* between indirect and direct heating is larger for Co than for Fe because of the smaller $G_{e,\mathrm{FM}-\mathrm{Au}}$, the electronic thermal conductance at the FM/Au interface. When we increase $G_{e,\mathrm{Co}-\mathrm{Au}}$ from 14 to 20 × 10^9^ W m^−2^ K^−1^, the deviation in *g* decreases from 24% to 7%: 10.3 × 10^17^ W m^−3^ K^−1^ for the indirect heating and 11 × 10^17^ W m^−3^ K^−1^ for the direct heating. Considering the uncertainties in the experiment and thermal modeling, we claim that the discrepancies of 7% and 24% for Fe and Co, respectively, are within the acceptable range. The sensitivities for the determination of *g* during the initial 20 or 40 ps are shown in Figure 4a,b,d,e, whereas the fitting results for the entire timescale of 1 ns are shown in Figure 4c,f. During the initial 4 ps with the direct heating configuration, TDTR data cannot be fitted with the thermal model because of the significant contribution of electrons on ∆*R*. Note that the peak of ∆*T*_e_ is about six times larger with the direct heating than with the indirect heating, as shown in Figure 1b. In addition, the direct heating produces a significant amount of non-thermal electrons on Au, whose effect on ∆*R* could be larger than that of thermalized electrons. Since thermal modeling analyzes ∆*T*_ph_, we ignore such a peak of TDTR in a few ps for the fitting process.

## 4. Discussion

Using the TDTR technique, we measured the transient increase in temperature of Au in the FM/Au bilayer with two different heating configurations: indirect (pump on FM) and direct (pump on Au) heating. By analyzing the dynamics of temperature response using thermal circuit modeling, we determined the electron-phonon coupling (*g*) parameters of Fe and Co: 8.8 × 10^17^ (indirect heating) and 9.4 × 10^17^ (direct heating) for Fe and 9.6 × 10^17^ (indirect heating) and 12.2 × 10^17^ (direct heating) for Co, all in a units of W m^−3^ K^−1^. These values are similar to the previous result of Ni of 8.6 × 10^17^ W m^−3^ K^−1^ [11]. Despite a large variation in the theoretical prediction, we demonstrated that the *g* parameters of all 3*d* transition FMs are in the order of 10^18^ W m^−3^ K^−1^. Our determination of the *g* parameters could be useful for future research for ultrafast transport of heat, charge, and spin in FM. In addition, we found that *g* values tend to be larger with direct heating than with indirect heating. As a possible origin for this discrepancy, we suspect the uncertainties on the assumptions in thermal modeling. For the direct heating, we assume an exponential distribution of initial energy with a characteristic length of 121.3 nm, considering the ballistic transport in Au. Such a simple exponential distribution may not accurately describe the effect of non-thermal electrons. Since the complexity of non-thermal electrons, which is difficult to describe in diffusive thermal modeling, is suppressed in indirect heating, we expect that the *g* determination from the indirect heating is more accurate than that from the direct heating. The indirect heating configuration in a bilayer can be applied to various metals to determine the *g* parameters.

## Figures and Tables

**Table 1:** Intrinsic properties included in the thermal circuit model.

	Au	Fe	Co	Sapphire
$c_{\mathrm{total}}$ ($10^{6}{\;J\;m}^{-3}{\;K}^{-1}$)	2.5 ^1^	3.5 ^1^	3.76 ^1^	3.08 ^1^
$c_{e}/T_{e}$ (${J\;m}^{-3}\;K^{-2}$)	67.6 ^2^	705 ^2^	710 ^2^	
$c_{\mathrm{ph}}$ ($10^{6}{\;J\;m}^{-3}{\;K}^{-1}$)	2.47 ^3^	3.32 ^3^	3.55 ^3^	3.08 ^3^
$\Lambda_{e}/T_{e}$ (${W\;m}^{-1}K^{-2}$)	0.7 ^4^	0.17 ^5^	0.18 ^5^	
$\Lambda_{\mathrm{ph}}$ (${W\;m}^{-1}K^{-1}$)	2.5 ^6^	9 ^5^	5 ^5^	30 ^7^
g ($10^{17}{\;W\;m}^{-3}\;K^{-1}$)	0.22 ^8^	8.8–9.4 ^9^	9.6–12.2 ^9^	
$G_{e,\mathrm{FM}-\mathrm{Au}}$ ($10^{9}{\;W\;m}^{-2}\;K^{-1}$)		20 ^10^	14 ^10^	
$G_{\mathrm{ph},\mathrm{FM}-\mathrm{Au}}$ ($10^{9}{\;W\;m}^{-2}\;K^{-1}$)		1 ^11^	1 ^11^	
$G_{\mathrm{ph},\mathrm{FM}-\mathrm{sap}}$ ($10^{9}{\;W\;m}^{-2}\;K^{-1}$)		0.35 ^9^	0.6 ^9^	
n	0.15 + 4.8j ^12^	2.9 + 3.33j ^13^	2.46 + 4.75j ^13^	1.76 ^14^
dn/dT ($10^{-4}\;K^{-1}$)	2 ^15^			

^1^ Reference [30]; ^2^ Reference [31]; ^3^ obtained by *c*_total_—*c*_e_ at 300 K; ^4^ Wiedemann-Franz law calculation with a measured electrical conductivity; ^5^ Reference [32]; ^6^ Reference [33]; ^7^ Reference [34]; ^8^ References [8,16,17]; ^9^ Determined values in this work; ^10^ Wiedemann-Franz law calculation with interfacial electrical resistances reported [24]; ^11^ Reference [25]; ^12^ Reference [35]; ^13^ Reference [36]; ^14^ Reference [37]; ^15^ Reference [29].

## Data Availability

Derived data supporting the findings of this study are available from the corresponding author on request.
